# A High-Protein Diet Promotes Atrial Arrhythmogenesis via Absent-in-Melanoma 2 Inflammasome

**DOI:** 10.3390/cells13020108

**Published:** 2024-01-05

**Authors:** Jia Song, Jiao Wu, Dexter J. Robichaux, Tingting Li, Shuyue Wang, Maria J. Arredondo Sancristobal, Bingning Dong, Dobromir Dobrev, Jason Karch, Sandhya S. Thomas, Na Li

**Affiliations:** 1 Department of Medicine, Section of Cardiovascular Research, Baylor College of Medicine, Houston, TX 77030, USAmajoarredondo@outlook.com (M.J.A.S.); 2 Department of Medicine, Section of Nephrology, Houston, TX 77030, USA; 3 Department of Integrative Physiology, Baylor College of Medicine, Houston, TX 77030, USAdobromir.dobrev@uk-essen.de (D.D.);; 4 Department of Medicine, Section of Gastroenterology, Baylor College of Medicine, Houston, TX 77030, USA; 5 Institute of Pharmacology, University Duisburg-Essen, 45147 Essen, Germany; 6 Department of Medicine, Montreal Heart Institute, Université de Montréal, Montréal, QC H1T 1C8, Canada; 7 Michael E. Debakey VA Medical Center, Houston, TX 77030, USA

**Keywords:** atrial fibrillation, high-protein diet, Absent-in-Melanoma, inflammasome, mitochondria

## Abstract

High-protein diets (HPDs) offer health benefits, such as weight management and improved metabolic profiles. The effects of HPD on cardiac arrhythmogenesis remain unclear. Atrial fibrillation (AF), the most common arrhythmia, is associated with inflammasome activation. The role of the Absent-in-Melanoma 2 (AIM2) inflammasome in AF pathogenesis remains unexplored. In this study, we discovered that HPD increased susceptibility to AF. To demonstrate the involvement of AIM2 signaling in the pathogenesis of HPD-induced AF, wildtype (WT) and *Aim2^−/−^* mice were fed normal-chow (NC) and HPD, respectively. Four weeks later, inflammasome activity was upregulated in the atria of WT-HPD mice, but not in the *Aim2^−/−^*-HPD mice. The increased AF vulnerability in WT-HPD mice was associated with abnormal sarcoplasmic reticulum (SR) Ca^2+^-release events in atrial myocytes. HPD increased the cytoplasmic double-strand (ds) DNA level, causing AIM2 activation. Genetic inhibition of AIM2 in *Aim2^−/−^* mice reduced susceptibility to AF, cytoplasmic dsDNA level, mitochondrial ROS production, and abnormal SR Ca^2+^-release in atrial myocytes. These data suggest that HPD creates a substrate conducive to AF development by activating the AIM2-inflammasome, which is associated with mitochondrial oxidative stress along with proarrhythmic SR Ca^2+^-release. Our data imply that targeting the AIM2 inflammasome might constitute a novel anti-AF strategy in certain patient subpopulations.

## 1. Introduction

Atrial fibrillation (AF) is the most common tachyarrhythmia observed in the clinic. AF can be promoted by ectopic firing and maintained by a reentrant substrate [1,2,3]. Despite considerable advancements in AF treatment and management, the recurrence and persistence of AF remain a clinical challenge for many patients [4,5,6]. The overall incidence and prevalence of AF is increasing globally [7]. Recently, AF prevention by targeting modifiable risk factors has shown promising therapeutic potential [8]. A high-protein diet (HPD) has been advocated for decades as a means of weight loss and preventing obesity-related metabolic derangements [9]. While HPD provides certain metabolic advantages, such as improved insulin sensitivity and reduced fatty liver disease, recent studies have uncovered that dietary protein may contribute to atherosclerosis and lesion complexity, and elevate the risk of chronic kidney disease [10,11,12]. It has been suggested that HPD may further the progression of coronary artery disease by increasing lipid deposition and inflammation, both of which are associated with AF development [13]. The direct link between HPD and AF pathogenesis has not been previously investigated and was the primary objective of this study.

The role of innate inflammatory signaling in AF pathogenesis has been recognized in recent years [13]. We have previously shown that activation of the ‘NLR family pyrin domain containing 3′ (NLRP3) inflammasome signaling enhances atrial arrhythmogenesis in various contexts, such as obesity, post-surgical procedures, and chronic kidney disease [14,15,16]. Although various inflammasome signaling pathways have been identified in innate immune cells, the potential role of the Absent-in-Melanoma 2 (AIM2) inflammasome in atrial arrhythmogenesis has not yet been investigated. AIM2 is a cytoplasmic sensor that can be activated by double-stranded DNA (dsDNA) originating from foreign pathogens or damaged cellular organelles, such as mitochondrial DNA (mtDNA) [17,18,19]. Upon sensing, AIM2 recruits ‘apoptosis-associated speck-like protein containing a CARD’ (ASC) and pro-caspase-1 to form the AIM2 inflammasome, subsequently promoting caspase-1 autocleavage and eliciting inflammatory responses via cytokine maturation [20,21]. Emerging evidence reveals that activation of the AIM2 inflammasome is involved in the development of cardiovascular diseases, such as atherosclerosis, myocardial infarction, aortic aneurysm, and heart failure [22,23,24]. It has been reported that AIM2 inflammasome activation contributes to chronic inflammation in heart failure, with necrotic DNA potentially serving as a major trigger for AIM2 inflammasome activation in vivo [23]. In this study, our findings demonstrate that HPD can activate the AIM2 inflammasome through altered mitochondrial function, thereby enhancing atrial arrhythmogenesis.

## 2. Materials and Methods

### 2.1. Animal Studies

Animal studies were performed according to the protocols approved by the Institutional Animal Care and Use Committee of Baylor College of Medicine and conformed to the Guide for the Care and Use of Laboratory Animals published by the US National Institutes of Health (NIH Publication No. 85-23, revised 1996). Whole-body *Aim2* gene-trap mice (*Aim2^−/−^*) [25] were purchased from the Jackson Laboratory (Strain #013144, Bar Harbor, ME, USA) and have been backcrossed with C57BL/6J mice more than six times, ensuring that both the wildtype (WT) and *Aim2^−/−^* mice were on the same genetic background. Apart from the genotyping protocol, the loss of *Aim2* transcripts in the atria of *Aim2^−/−^* mice was also confirmed with reverse-transcription (RT)-PCR using atrial RNA samples (Supplementary Figure S1). The primer sequences were as follows (5′ > 3′): *Aim2*—forward CACACTCGACGTGGCAGATAGGAC, reverse CAGCACCGTGACAACAAGTGG; *Rpl7*—forward ATCATCTGCATGGAGGATCTAAT, reverse TCATCCGTCTAATAAGCCTGTTT. Six-to-eight-week-old wildtype (WT) and *Aim2^−/−^* mice were subjected to either normal chow (NC) or HPD (40% protein diet, ENVIGO Company, Indianapolis, IN, USA), respectively, for 4 weeks before in vivo and ex vivo studies. NC was composed of 21% kcal protein, 64% kcal carbohydrate, and 15% kcal fat, whereas HPD was composed of 42% kcal protein, 44% kcal carbohydrate, and 13.1% kcal fat. Each group included an approximately equal number of male and female mice.

### 2.2. Serum Creatinine

Creatinine levels in sera were measured with the Creatinine (serum) Colorimetric Assay kit (Cayman Chemicals, Ann Arbor, MI, USA, 7000460). Briefly, 15 µL of sera sample was loaded into duplicate wells, followed by adding the Creatinine Reaction buffer and Creatinine (serum) Color Reagent. Absorbance at 490–500 nm was measured immediately after the Color Reagent buffer was added and repeated 7 min later.

### 2.3. Glucose and Insulin Tolerance Tests

Glucose (1.5 g/kg body mass, i.p.) and insulin (1.0 unit/kg body mass, i.p.) tolerance tests (GTT, ITT) were conducted to determine blood glucose clearance and insulin response, respectively. Mice underwent a 16 or 4 h fasting period, respectively, prior to GTT or ITT. For GTT, glucose was intraperitoneally injected, and tail vein blood was collected at 0, 30, 60, 90, and 120 min intervals to assess blood glucose concentration. For ITT, insulin was intraperitoneally injected, and tail vein blood was collected at 0, 15, 30, 60, and 90 min intervals to measure blood glucose concentration.

### 2.4. Western Blotting

Western blots were prepared using the Bio-Rad Mini Trans-Blot^®^ system. Briefly, protein from homogenized atrial tissue (60 µg) was separated on an acrylamide gel before transfer to Immobilon^®^-FL Transfer membrane (PVDF) (Millipore, Burlington, MA, USA, IPFL00010). The PVDF membrane was blocked with Intercept Blocking Buffer (Li-COR Biosciences, Lincoln, NE, USA, 927-60001) before being probed with antibodies. The primary antibodies included ASC (1:1000, Cell Signaling Technology, Danvers, MA, USA, 2118), Caspase-1 (1:1000, Biovision, Milpitas, CA, USA, #3019-100), IL-1β (1:1000, Abcam, Waltham, MA, USA, ab9722), CD68 (1:1000, Abcam, Waltham, MA, USA, ab201973), F4/80 (D2S9R) (1:1000, Cell Signaling Technology, Danvers, MA, USA, 70076), GAPDH Rabbit mAb (1:1000, Cell Signaling Technology, Danvers, MA, USA, 5174), and GAPDH Mouse mAb (1:1000, Cell Signaling Technology, Danvers, MA, USA, 97166). The primary antibody was added to the membrane and incubated overnight at 4 °C. After washing three times with TBST, the secondary antibodies were added according to the species of the primary antibodies, including goat anti-mouse IgG (H+L) Alexa Fluor™ Plus 800 (1:10,000, Invitrogen, Waltham, MA, USA, A32730); goat anti-rabbit IgG (H+L) Alexa Fluor™ Plus 680 (1:10,000, Invitrogen, Waltham, MA, USA, A32734); goat anti-mouse IgG (H+L) Alexa Fluor™ Plus 680 (1:10,000, Invitrogen, Waltham, MA, USA, A32729); and goat anti-rabbit IgG (H+L) Alexa Fluor™ Plus 800 (1:10,000, Invitrogen, Waltham, MA, USA, A32735). The Odyssey system (Li-COR Biosciences, Lincoln, NE, USA, Serial NO.380) was used to develop the blots. The Image J (Version 1.53a) densitometry function was used to analyze the resulting bands.

### 2.5. Circulating Cytokines and Biomarkers

Serum was collected from WT and *Aim2^−/−^* mice after 4 weeks of NC or HPD treatment, respectively. IL-1β and IL-18 levels were determined according to the instructions provided by the ELISA kits (Mouse IL-1β/IL-1F2 Quantikine ELISA Kit, R&D Systems, Minneapolis, MN, USA, MLB00C; Mouse IL-18 ELISA kit, Medical & Biological Laboratories, Naka-Ku, Nagoya, Japan, CODE No.7625).

### 2.6. In Vivo Electrophysiology Studies

Programmed electrical stimulation (PES) was performed to determine cardiac electrophysiological differences among treatment groups using the previously published protocols [14,16,26,27]. Briefly, mice were anesthetized with a 1.5% isoflurane/oxygen mixture. A 1.1F octapolar catheter (EPR-800, Millar Instruments, Houston, TX, USA) was inserted into the heart through the right jugular vein. The iWorx Data Acquisition System (iWorx System, Dover, NH, USA, iWorx-Bio8) was used to record surface ECG and intracardiac electrograms, and bipolar pacing was performed to assess sinus node recovery time (SNRT), atrial effective refractory period (AERP), and atrioventricular node effective refractory period (AVNERP). The inducibility of AF was determined using an overdriving pacing protocol to induce AF. Mice underwent the same atrial pacing protocol 3 times, and only mice in which AF (≥1 s) could be induced in at least 2 out of 3 attempts were considered AF-positive. The incidence of inducible AF was calculated as the percentage of the AF-positive mice divided by the total number of mice studied. For quantification of AF duration, we capped the duration at 5 min for any episodes longer than 5 min. 

### 2.7. Echocardiography

Systolic cardiac function was assessed by echocardiography using VisualSonics Vevo F2 Ultrasound (FujiFilm VisualSonics, Toronto, ON, Canada) as described previously [14,16,26]. Briefly, mice were anesthetized using 1.5–2% isoflurane mixed with 100% O_2_ and placed on a heated platform, with their four limbs taped to ECG electrodes. Body temperature was monitored via a rectal temperature probe and maintained at 37 °C. Left ventricular (LV) function and structure were assessed using M-mode images acquired from the parasternal short-axis views at mid-papillary level. The left atrium (LA) area was measured using B-mode images from the parasternal long-axis views.

### 2.8. Histology

A whole heart was harvested from the mouse, perfused with 4% KCL, fixed in 10% buffered formalin for 24–48 h, and embedded in paraffin wax. Masson trichrome staining was performed on 6 μm sections. Images were acquired using a Zeiss microscope, and the percentage of fibrosis was quantified using Image J (Version 1.53a).

### 2.9. Calcium Imaging

Atrial myocytes were isolated as described [14,16,26,27]. Mouse hearts, harvested during anesthesia, were cannulated and perfused in Langendorff mode with Ca^2+^-free Tyrode solution for 1–3 min at 37 °C, followed by 0.022 mg/mL Liberase (TH Research Grade, Sigma-Aldrich, St. Louis, MO, USA, 5401151001) in Ca^2+^-free Tyrode at 37 °C until digestion was complete. Typically, the digestion process took between 25–30 min, depending on the flow rate of the efflux buffer. After digestion, the heart was perfused with 5 mL of KB solution containing KCl (90 mmol/L), K_2_HPO_4_ (30 mmol/L), MgSO_4_ (5 mmol/L), pyruvic acid (5 mmol/L), β-hydroxybutyric acid (5 mmol/L), creatine (5 mmol/L), taurine (20 mmol/L), glucose (10 mmol/L), EGTA (0.5 mmol/L), and HEPES (5 mmol/L), adjusted to pH 7.2. The atria were dissected, minced in KB solution, and filtered through a 210 μm polyethylene mesh. The cells were stored in KB solution at room temperature and then incubated with Cal-520 (4 μM) for 30 min at room temperature prior to imaging in normal Tyrode’s solution containing 1.8 mmol/L Ca^2+^. Confocal (LSM 510, Carl Zeiss, Oberkochen, Germany) fluorescence images were recorded in line-scan mode. To minimize the impact of EGTA and high extracellular K^+^ on Ca^2+^ dynamics, the extracellular KB buffer was replaced with a normal Tyrode solution before adding the cells to the recording chamber. Afterward, the cells were also perfused with normal Tyrode solution both before and during Ca^2+^ imaging. Atrial myocytes that faithfully follow the field stimulation during acclimation are chosen for recording. Once steady-state Ca^2+^ transients were observed during 1 Hz pacing (10 V), pacing was stopped, and Ca^2+^ sparks were counted using SparkMaster. To assess the sarcoplasmic reticulum (SR) Ca^2+^ load, caffeine (1 mmol/L) was applied.

### 2.10. Real-Time PCR (qPCR)

RNA was extracted from atrial tissue using the Trizol reagent (Invitrogen, Waltham, MA, USA). For the reverse transcription process, a total of 1 μg of RNA was used in a final reaction volume of 20 μL, which included 4 μL of iScript Reverse Transcription Supermix (Bio-Rad, Hercules, CA, USA, 1708841). Following reverse transcription, the cDNA was diluted threefold. PCR was conducted in triplicate through 40 cycles, using 1 μL of the cDNA volume in a total 20 μL reaction mixture containing 10 μL of PowerUP SYBR Green Master Mix (Thermo Fisher, Waltham, MA, USA, A25742). Real-time PCR analysis for mRNA quantification was performed on the QuantStudio5 system (Thermo Fisher, Waltham, MA, USA). The thermocycler conditions comprised 40 cycles, including denaturation at 95 °C for 15 s and an annealing/extension step at 60 °C for 60 s. The ΔCT method was used to quantify all relative mRNA levels, with *Rpl7* as the reference and internal standard. The primer sequences were as follows (5′ > 3′): *Rpl7*—forward ATCATCTGCATGGAGGATCTAAT, reverse TCATCCGTCTAATAAGCCTGTTT; *Nppa*—forward CACAGATCTGATGGATTTCAAGA, reverse CCTCATCTTCTACCGGCATC; *Myh6*—forward TGCTGAGGGAACAGTATGAA, reverse TCTGTATGGCATCCGTCTC; *Myh7*—forward ACAGAGGAAGACAGGAAGAACC, reverse GCTTGTTGACCTGGGACTC.

### 2.11. Immunofluorescence Staining to Detect Cytosolic DNA

Hearts were embedded in paraffin following a standard protocol and sectioned to a thickness of 6 μm. After deparaffinization, heat-induced epitope retrieval was performed using a pressure cooker. Nonspecific staining was minimized by blocking with 10% donkey serum. The sections were then incubated with primary antibodies overnight at 4 °C, washed with PBS, and subsequently incubated with secondary antibodies. The primary antibodies used include anti-TOMM20 (1:200, Abcam, Waltham, MA, USA, ab-78547) and anti-dsDNA (1:200, Abcam, Waltham, MA, USA, ab-27156). The secondary antibodies used are Alexa Fluor™ 568 donkey anti-mouse IgG (H+L) (1:200, Invitrogen, Waltham, MA, USA, A10037) and Alexa Fluor™ 488 donkey anti-rabbit IgG (H+L) (1:200, Invitrogen, Waltham, MA, USA, A32790). Nuclei were counterstained with 4′,6-diamidino-2-phenylindole (DAPI). As negative controls, slides of sections were also incubated with secondary antibodies alone. Tissue sections were examined using a confocal microscope (LSM 510, Carl Zeiss, Oberkochen, Germany).

### 2.12. Cytoplasmic Fractionation to Detect Cytosolic DNA

Following atrial myocyte isolation as described above, a single-cell suspension was resuspended in 1 mL of PBS and transferred to a 1.5 mL Eppendorf tube. After a brief spin in the centrifugation, the supernatant was removed. Five hundred microliters of hypotonic lysis buffer (20 mM HEPES, 10 mM KCL, 2 mM MgCl_2_, 1 mM EDTA, 1 mM EGTA, 1 mM DTT, and protease inhibitors) was added to the tube, then incubated on ice for 15 min with occasional vortexing. Fifteen minutes later, cells in the tube were sheared through a 23 g needle with a 1 mL syringe and then incubated for an additional 15 min on ice. The tube was subsequently centrifuged, and the supernatant was transferred to a new tube. The pellet, containing nuclei and cell debris, was discarded. The supernatant, which contained cytosolic DNA and mitochondria, was transferred to a new tube. The tube was then centrifuged at 4 °C, 15 min, and 15,000× *g* to remove mitochondria. The clarified supernatant (containing cytosolic DNA) was transferred to a new tube. dsDNA was directly quantified by the Qubit dsDNA High Sensitivity Assay (Thermo Fisher, Waltham, MA, USA, Q33232) following the manufacturer’s instructions. Protein concentration was determined using the BCA Assay. The final data was calculated as cytoplasmic DNA normalized to protein concentration.

### 2.13. Reactive Oxygen Species (ROS) Detection

Atrial myocytes were isolated as described above. Atrial myocytes are stained with MitoSOX™ Mitochondrial Superoxide Indicators (Invitrogen, Waltham, MA, USA, M36008) following the instruction. The presence of ROS was detected using a confocal microscope (LSM 510, Carl Zeiss, Oberkochen, Germany).

### 2.14. Statistical Analysis

Numerical data were presented as the mean ± SEM. Statistical analyses were performed using GraphPad Prism 9.0. Normality was assessed using the Kolmogorov–Smirnov test. A Student’s *t*-test was used to compare data between two groups maintaining normal distributions. The Mann–Whitney test was used for data where normality cannot be assumed. ANOVA followed by Sidak’s or Dunnett’s T3 test was used for multiple comparisons. The Kruskal–Wallis test, followed by the Dunn test, was used for multiple comparisons of nonparametric data. Fisher’s exact test was used to compare categorical data. A nested one-way ANOVA followed by Sidak’s test was used for data from biological replicates with repeated measurements. A *p*-value of <0.05 was considered statistically significant.

## 3. Results

### 3.1. HPD Displayed Mild Beneficial Effects on Body Weight and Glucose Tolerance

**Figure 1:**
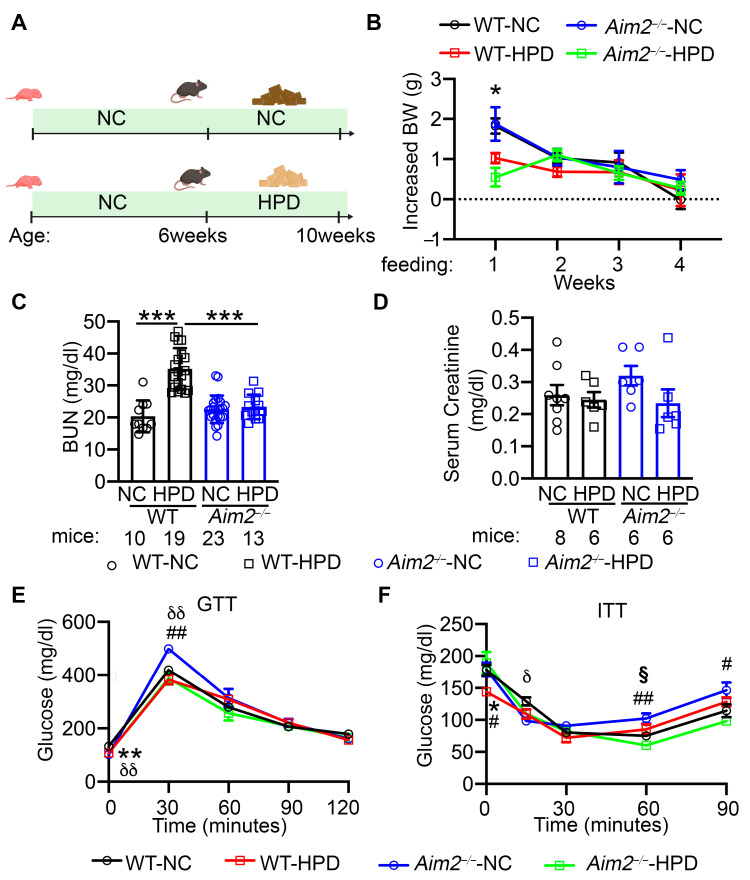
High-protein diet (HPD) displayed salutary effects on body weight and glucose tolerance. (**A**) Group design of WT and *Aim2^−/−^* mice received normal chow (NC) or HPD, respectively. Created with BioRender.com. (**B**) Weekly body weight (BW) gain. N = 8 mice/group. (**C**) Blood urea nitrogen level (BUN) levels. (**D**) Serum creatinine levels. (**E**,**F**) Plasma level of glucose during glucose-tolerance test (GTT, **E**) and insulin-tolerance test (ITT, **F**) in WT and *Aim2^−/−^*-mice after 4 weeks of feeding with NC or HPD, respectively. In (**B**,**C**), * *p* < 0.05, *** *p* < 0.001. In (**E**,**F**), * *p* < 0.05, ** *p* < 0.01, WT-NC vs. WT-HPD; δ *p* < 0.05, δδ *p* < 0.01, WT-NC vs. *Aim2^−/−^* -NC; # *p* < 0.05, ## *p* < 0.01, *Aim2^−/−^*-NC vs. *Aim2^−/−^*-HPD; § *p* < 0.05, WT-HPD vs. *Aim2^−/−^*-HPD. *p*-values were determined by Welch ANOVA and Dunnett’s T3 multiple comparisons test in (**B**,**C**,**E**), and Welch ANOVA and Dunnett’s T3 multiple comparisons test and Kruskal–Wallis with Dunn’s multiple comparisons test in (**F**).

Previously, we showed that NLRP3 inflammasome activation in atrial tissue is involved in AF pathogenesis and mediates atrial arrhythmogenesis in the context of obesity or chronic kidney disease [14,16,27]. To determine the impact of HPD on atrial arrhythmogenesis and whether the NLRP3 inflammasome plays a role, we first subjected WT and *Nlrp3^−/−^* to NC and HPD, respectively, for 4 weeks. Interestingly, HPD enhanced AF inducibility in WT mice, but genetic ablation of NLRP3 did not prevent HPD-induced AF susceptibility (Supplementary Figure S2). As other inflammasome pathways have not been studied in the context of AF previously, we investigated whether the AIM2 inflammasome could be involved in HPD-induced atrial arrhythmogenesis. For this, we subjected age-matched WT and *Aim2^−/−^* mice to NC or HPD, respectively, for 4 weeks (Figure 1A). At 6 weeks of age, there was no difference in body weight (BW) between WT and *Aim2^−/−^* mice. After the first week of HPD feeding, both WT and *Aim2^−/−^* mice gained less BW compared to their NC-fed controls (WT-NC vs. WT-HPD, *p* < 0.05; *Aim2^−/−^*-NC vs. *Aim2^−/−^*-HPD, *p* = 0.051, Figure 1B). However, the differences in BW gain disappeared during the following 3 weeks of feeding. Given that HPD may lead to a higher level of urea and other nitrogenous waste products [28], we assessed the kidney function after 4 weeks of feeding by measuring the blood urea nitrogen levels (BUN) [29,30] and serum creatinine levels in the four groups of mice. We found significantly higher BUN levels in WT-HPD mice than in WT-NC mice (*p* < 0.001, Figure 1C). Interestingly, BUN levels were reduced in *Aim2^−/−^*-HPD mice compared to WT-HPD mice (*p* < 0.001, Figure 1C). Despite these changes in BUN levels, all four groups of mice displayed similar serum creatinine levels (Figure 1D), indicating that kidney function was not affected by 4 weeks of HPD. To determine whether HPD or AIM2 deficiency altered glucose metabolism, we performed GTT and ITT after 4 weeks of feeding. For GTT, both WT-HPD and *Aim2^−/−^*-HPD mice showed a mild reduction in glucose levels at the 0 and 30 min sampling points compared to their respective NC-fed controls, although the area under the curve (AUC) was comparable among the four groups (Figure 1E, Supplementary Figure S3A,C). For ITT, HPD-fed *Aim2^−/−^* mice had a slightly better response to insulin challenge compared to the NC-fed *Aim2^−/−^* mice. The AUC of ITT was also comparable among the four groups (Figure 1F, Supplementary Figure S3B,D). In line with previous reports [31], these results suggest that one month of HPD feeding can slightly improve glucose tolerance, and AIM2 inhibition may have a mild effect on glucose homeostasis in mice.

### 3.2. AIM2 Inhibition Reduced the HPD-Induced Inflammasome Activity

**Figure 2:**
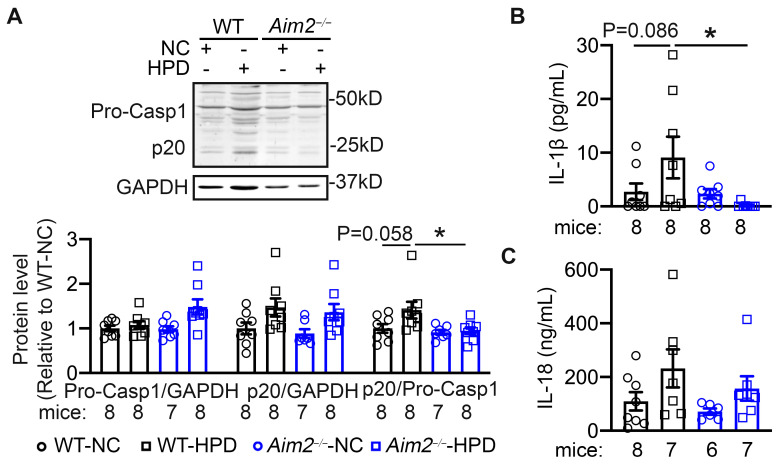
Inhibition of AIM2 prevents the high-protein diet (HPD)-induced caspase-1 activation. (**A**) Representative Western blots of caspase-1 and quantification of precursor caspase-1 (Pro-Casp1) and cleaved caspase-1 (p20). (**B**,**C**) Serum levels of IL-1β. (**B**) and IL-18 (**C**) * *p* < 0.05, *p*-values determined by ordinary one-way ANOVA with Sidak’s multiple comparisons test in (**A**) and Kruskal–Wallis with Dunn’s multiple comparisons test in (**B**).

To determine whether HPD activates the AIM2 inflammasome in mouse atria, Western blots were performed with atrial tissue of WT-NC, WT-HPD, *Aim2^−/−^*-NC, and *Aim2^−/−^*-HPD mice. We found that, while the protein levels of precursor caspase-1 were unchanged between WT-NC and WT-HPD mice, the levels of cleaved caspase-1 (p20) were upregulated in WT-HPD compared to WT-NC mouse atria (Figure 2A). Consistently, the serum levels of IL-1β and IL-18 cytokines showed a trend to be higher in WT-HPD mice than in WT-NC mice (Figure 2B,C), revealing a low-degree and subclinical inflammation status. Genetic inhibition of AIM2 attenuated the increases in protein levels of p20 and the circulating levels of IL-1β and IL-18 cytokines in *Aim2^−/−^*-HPD mice (Figure 2A–C). These results suggest that HPD enhances the atrial inflammatory response, which was prevented by the genetic inhibition of AIM2.

### 3.3. AIM2 Inhibition Reduced the Susceptibility to HPD-Induced AF

**Figure 3:**
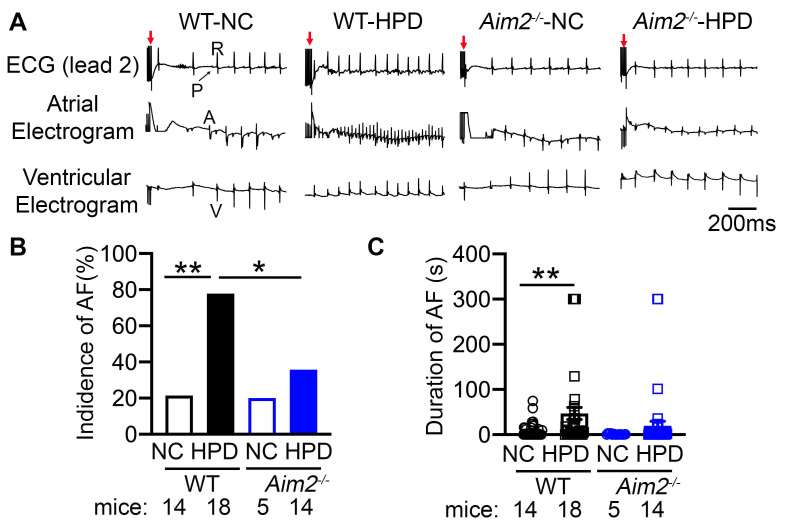
High-protein diet (HPD) enhances AF susceptibility via AIM2 inflammasome. (**A**) Representative recordings of lead-2 surface ECG and intracardiac electrograms in WT and *Aim2^−/−^* mice after 4 weeks’ feeding of NC or HPD, respectively. Red arrows indicate the end of the burst pacing protocol. ‘P’ and ‘R’ designate the P-wave and QRS complex on surface ECG, while ‘A’ and ‘V’ represent the atrial excitation and ventricular excitation on intracardiac electrograms, respectively. (**B**) The incidence of pacing-induced reproducible AF. (**C**) The duration of pacing-induced AF. * *p* < 0.05, ** *p* < 0.01. *p*-values determined by Fisher’s exact test in (**B**), and Kruskal–Wallis with Dunn’s multiple comparisons test in (**C**).

To determine whether HPD affects cardiac electrophysiology, we measured ECG parameters in four groups of mice after 4 weeks of feeding. PQ-, QRS-, and QTc-intervals, SNRT, and AERP parameters were all comparable among the four groups of mice, while only AVNERP was reduced in the WT-HPD group compared to the WT-NC group (Supplementary Table S1). To determine the impact of HPD on arrhythmogenesis, we performed PES to induce AF. Rapid atrial pacing induced AF in 77.8% of WT-HPD mice, whereas only 21.4% of WT-NC mice developed the pacing-induced AF (*p* < 0.01, Figure 3A,B). The average duration of all AF episodes was longer in WT-HPD mice than in WT-NC mice (*p* < 0.01, Figure 3C). Conversely, inhibition of AIM2 reduced the incidence and duration of AF in *Aim2^−/−^*-HPD (*p* < 0.05 vs. WT-HPD, Figure 3A,B). Importantly, there was no pacing-induced ventricular tachycardia in all groups of mice. These data suggest that HPD promotes the evolution of an arrhythmogenic substrate for AF, which requires the activation of the AIM2 inflammasome pathway.

### 3.4. HPD Promoted AF Independently from Structural Remodeling and Fibrosis in Atria

**Figure 4:**
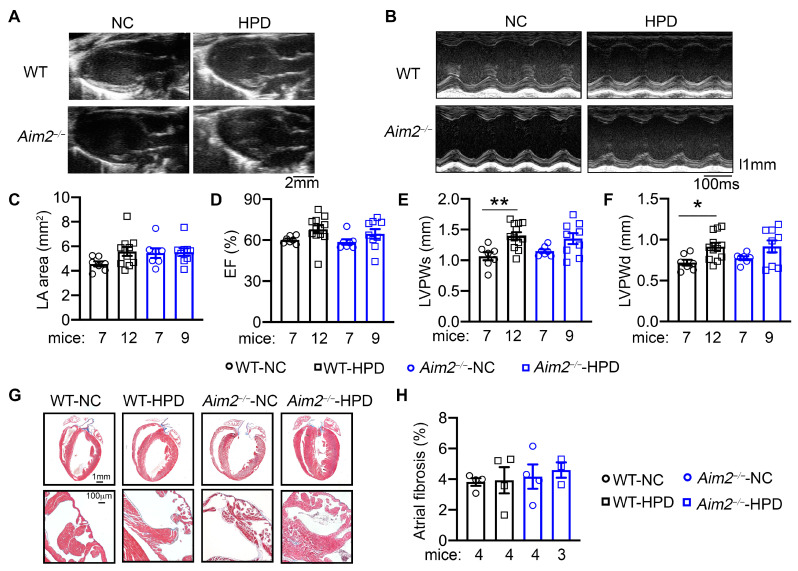
High-protein diet (HPD) promotes mild ventricular hypertrophy. (**A**) Representative long-axis echocardiography recording to assess the left atria (LA) area. (**B**) Representative M-mode echocardiography recordings in four groups of mice at the end of the 4-week feeding period. (**C**) Quantification of LA area. (**D**) Quantification of ejection fraction (EF%). (**E**,**F**) Quantification of LV posterior wall thickness during systole and diastole (LVPWs, (**E**); LVPWd, (**F**)). (**G**) Representative Masson Trichome’s staining in cardiac tissue. The second row of images indicated the left atria. (**H**) Quantification of the percentage of fibrosis in atrial tissue. * *p* < 0.05, ** *p* < 0.01. *p*-values were determined by Welch ANOVA and Dunnett’s T3 multiple comparisons test in (**E**,**F**).

To determine whether structural remodeling within the atria or ventricular dysfunction contributes to the enhanced AF susceptibility in the HPD model, we performed echocardiography analysis prior to the PES studies. We found that the left atrial (LA) dimensions were similar among the four groups (Figure 4A,C). Additionally, we found that all four groups of mice displayed similar left ventricular (LV) ejection fraction (EF%, Figure 4B,D), LV diameters (ESD, EDD), and LV anterior wall thickness (LVAWs, LVAWd) (Supplementary Table S2, Figure S4). Interestingly, LV posterior wall thickness during both systole and diastole (LVPWs and LVPWd) was increased in WT-HPD and *Aim2^−/−^*-HPD compared to their NC-fed controls (Figure 4E,F), indicating that 4-week HPD may promote physiological LV hypertrophy. However, the mRNA levels of hypertrophic makers (e.g., *Nppa*, *Myh7*, and *Mhy6*) were not significantly different among the four groups in the ventricle sample of the mice (Supplemental Figure S4). To further evaluate whether HPD promotes fibrotic remodeling, a known pro-AF substrate, histology, and Masson’s trichrome staining were performed. We found that atrial and ventricular fibrosis were comparable among the four groups (Figure 4G,H). Protein levels of the macrophage markers (CD68 and F4/80) in atria were also comparable among the four groups (Supplemental Figure S5). These results establish that HPD promotes the development of a pro-arrhythmic substrate for AF unrelated to structural and fibrotic remodeling of the atria.

### 3.5. Inhibition of AIM2 Reduced the Aberrant Diastolic Ca^2+^ Leak Associated with the HPD-Induced AF

**Figure 5:**
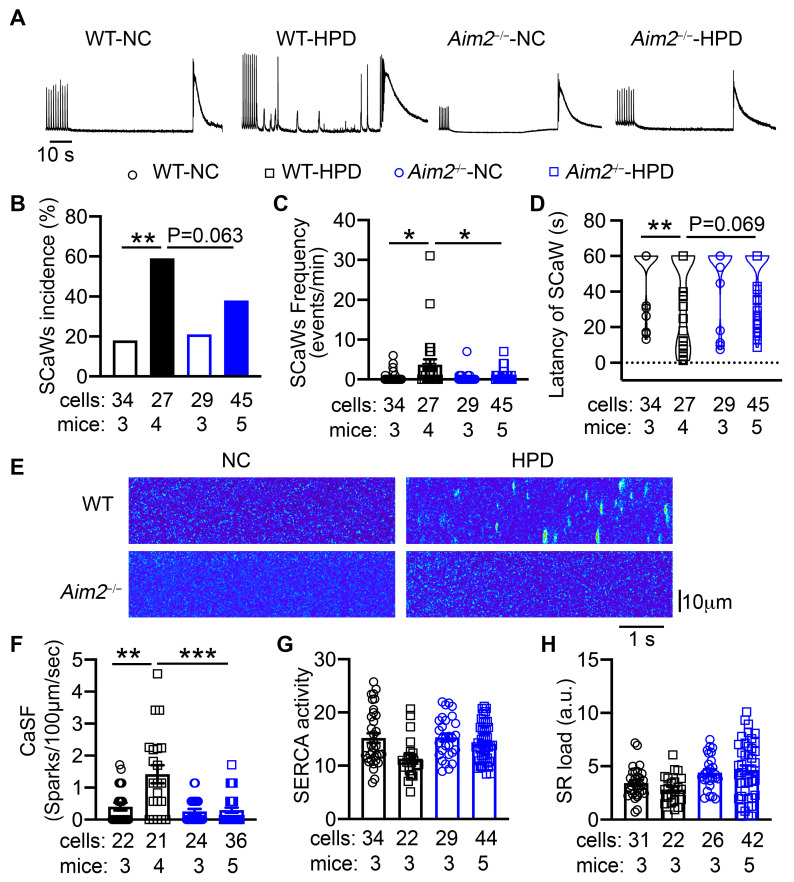
High-protein diet (HPD) promotes aberrant SR Ca^2+^ release events. (**A**) Representative traces of the 1 Hz pacing-induced Ca^2+^ transients (CaTs), followed by baseline recording and the caffeine (10 mmol/L)-induced CaTs in atrial myocytes from WT-NC, WT-HPD, *Aim2^−/−^*-NC, and *Aim2^−/−^*-HPD mice. (**B**) Incidence and (**C**) frequency of spontaneous Ca^2+^ waves (SCaWs). (**D**) The latency to the onset of SCaWs in atrial cardiomyocytes of 4 groups of mice. (**E**) Representative Ca^2+^ spark recordings. (**F**) Quantification of Ca^2+^ spark frequency (CaSF). (**G**) SERCA activity estimated as the decay of pacing-induced CaTs. (**H**) Estimation of sarcoplasmic reticulum (SR) Ca^2+^ load. * *p* < 0.05, ** *p* < 0.01, *** *p* < 0.001. *p*-values determined by Fisher’s exact test (one-tailed) in (**B**), Welch ANOVA with Dunnett’s T3 multiple comparisons test in (**C**), and Kruskal–Wallis with Dunn’s multiple comparisons test in (**D**), and nested one-way ANOVA with Sidak’s multiple comparisons text in (**F**).

To determine whether HPD promotes abnormal SR Ca^2+^ release events, we performed Ca^2+^ imaging studies in isolated atrial myocytes from four groups of mice. After 1 Hz pacing, the incidence of spontaneous Ca^2+^ waves (SCaWs) was significantly higher in the atrial myocytes from WT-HPD than those of WT-NC mice (*p* < 0.05, Figure 5A,B). The frequency of SCaWs was also greater in the atrial myocytes of WT-HPD than those of WT-NC mice (*p* < 0.05, Figure 5C). The latency to onset of SCaWs was shorter in the cells of WT-HPD mice (Figure 5D). Consistently, the frequency of Ca^2+^ sparks (CaSF), mediated by clusters of ryanodine receptor 2, was increased in the atrial myocytes of WT-HPD mice (*p* < 0.01 vs. WT-NC mice, Figure 5F). The activity of SERCA, estimated by the decay of pacing-induced Ca^2+^ transients, as well as the SR load, were unchanged among the four groups (Figure 5G,H). In contrast, atrial myocytes of *Aim2^−/−^*-HPD mice displayed reduced incidence and frequency of SCaWs, delayed latency to the onset of SCaWs, and less CaSF compared with the cells of WT-HPD mice (Figure 5). These results suggest that HPD can promote abnormal Ca^2+^ release-mediated triggered activity, thereby enhancing atrial arrhythmogenesis, which can be attenuated by the inhibition of the AIM2 inflammasome.

### 3.6. HPD Upregulates the AIM2 Inflammasome by Causing Mitochondrial Dysfunction

**Figure 6:**
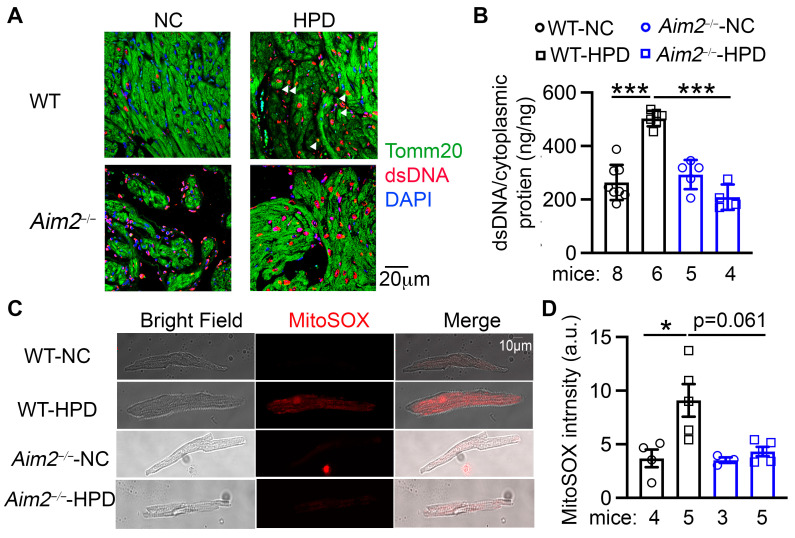
High-protein diet (HPD) increases cytoplasmic dsDNA content by promoting mitochondrial damage. (**A**) Immunostaining of dsDNA and Tomm20 (a mitochondrial marker) in the atrial tissue of four groups of mice. White arrows point to the cytoplasmic dsDNA signal that overlapped with Tomm20 but not with DAPI. (**B**) Cytoplasmic dsDNA levels in atrial lysates of WT and *Aim2^−/−^* mice subjected to NC or HPD, respectively. (**C**) Representative images of MitoSOX staining in atrial myocytes. (**D**) Aggregated analysis of MitoSOX intensity. The number of cells analyzed in each group is as follows: 55 in WT-NC, 43 in WT-HPD, 34 in *Aim2^−/−^*-NC, and 26 in *Aim2^−/−^*-HPD. * *p* < 0.05, *** *p* < 0.001. *p*-values were determined by ordinary one-way ANOVA with Sidak’s multiple comparisons test in (**B**) and Kruskal–Wallis with Dunn’s multiple comparisons test in (**D**).

Finally, to determine how HPD enhances the AIM2 inflammasome, we examined the levels of dsDNA in atria, a major activator of the AIM2 inflammasome. We first conducted immunofluorescence staining to examine the localization of dsDNA (Figure 6A). Compared with the atrial tissue of WT-NC mice, WT-HPD atria exhibited a markedly increased level of dsDNA, some of which co-localized with the mitochondrial marker Tomm20 (Figure 6A). We then measured the cytoplasmic dsDNA content in atrial myocytes using a dsDNA hypersensitive kit. We found that the cytoplasmic dsDNA content was increased in the atria of WT-HPD mice compared with that of WT-NC mice (*p* < 0.001, Figure 6B). These data suggest that HPD may increase cytoplasmic mitochondrial DNA (mtDNA) content, thereby activating the AIM2 inflammasome pathway. The increases in the cytoplasmic dsDNA and mtDNA content were reversed in the *Aim2^−/−^*-HPD group (Figure 6A,B), indicating a potential feedforward feedback loop between HPD-mediated increases in dsDNA and AIM2 activation. To determine whether the increased mtDNA release is a result of mitochondrial damage, we assessed levels of mitochondrial reactive oxidative species (mtROS) by staining atrial myocytes with MitoSOX^TM^ Red. We observed that mtROS levels were significantly increased in the atrial myocytes of WT-HPD mice compared to those of WT-NC mice (Figure 6C,D and Supplementary Figure S6). Interestingly, mtROS levels in the atrial myocytes of *Aim2^−/−^*-HPD mice were also markedly decreased compared with those from WT-HPD mice (Figure 6C,D and Supplementary Figure S6). These results suggest that HPD-induced mitochondrial stress may activate the atrial AIM2 inflammasome.

## 4. Discussion

In the present study, using the custom-made protein-enriched diet (40% protein content), we observed that 4-week short-term HPD can mildly improve glucose tolerance, in line with previous findings [31]. Our results also present the first evidence that HPD enhances atrial arrhythmogenesis by promoting abnormal diastolic Ca^2+^ release events, which depend on the activity of the AIM2 inflammasome in the atria. Importantly, we demonstrate that HPD activates the AIM2 inflammasome by causing mitochondrial stress. To our knowledge, this is the first study to identify an adverse effect of HPD on cardiac electrophysiology and to establish that the AIM2 inflammasome contributes to HPD-mediated atrial arrhythmogenesis.

To date, the association between HPD and cardiovascular diseases has not been well established. While protein-enriched diets and supplementary dietary proteins are widely accepted for weight management and metabolic profile improvement, the health benefits of macronutrients, often derived from dietary protein, continue to be a subject of debate [14,32]. For instance, HPD may exacerbate the progression of coronary artery disease by promoting lipid deposition and activating inflammatory pathways, thereby increasing AF susceptibility. Recent studies also suggest that HPD intake can cause intraglomerular hypertension and increase the risk of chronic kidney disease [11,12], which in turn is associated with a higher risk of AF as a result of elevated circulating IL-1β levels [16]. In our study, the HPD-fed mice exhibit normal serum creatinine levels, in spite of elevated BUN levels. This suggests that kidney function remains compensated after 4 weeks of HPD. While there is no apparent structural remodeling in the atria, the left ventricular posterior wall was thickened in HPD-fed mice. Whether this hypertrophic response has physiological or pathophysiological consequences requires further examination.

Our prior research has established that the NLRP3 inflammasome plays a critical role in AF development in obesity or chronic kidney disease models, serving as a mechanistic link between these risk factors and increased AF risk [14,16,27]. As a known member of the inflammasome family, the AIM2 inflammasome has been implicated in promoting ischemic cardiomyopathy, the progression of atherosclerosis, aortic aneurysms, and ischemic strokes [22,23]. In this study, we reveal that global genetic inactivation of AIM2 prevents the HPD-induced increase in AF susceptibility, providing the first evidence that atrial AIM2 inflammasome activity could also be involved in AF pathogenesis, particularly in the context of HPD.

AIM2 is activated upon binding to a minimum of 250–300 bp of dsDNA in a non-sequence-specific manner [33,34]. Once activated, AIM2 interacts with the adaptor protein ASC and pro-caspase-1, forming the inflammasome complex and leading to caspase-1 autocleavage. Mature caspase-1 can then promote the maturation of IL-1β and IL-18 [35,36]. Our findings show that HPD elevates atrial levels of mature caspase-1, which can be prevented by the genetic inhibition of AIM2. During sterile inflammation, damaged cellular organelles represent a significant source of danger signals. dsDNA is usually confined within the nucleus or mitochondria, and the presence of cytoplasmic dsDNA is an indication of cellular stress [37]. In the HPD model, the increased level of cytoplasmic dsDNA is associated with increased mitochondrial ROS production, typically indicating a state of mitochondrial stress and dysfunction. Mitochondria play an essential role in cardiomyocyte ATP production and are also a primary site of ROS generation [38,39]. Excessive protein intake has been linked to oxidative damage in the mouse pancreas and rat brain by reducing antioxidant systems such as superoxide dismutase. In contrast, protein restriction lowers mitochondrial ROS and mtDNA levels, as well as lipid damage in the rat liver [40]. These studies, together with our current findings, suggest that HPD consumption can distress mitochondria and cause the subsequent release of mtDNA, thereby activating the AIM2 inflammasome. Since oxidative stress is known to be a factor in the development of AF in patients, our study adds a new dimension to the understanding of AF pathogenesis, implicating enhanced AIM2 inflammasome signaling due to oxidative stress. Future studies should explore whether targeting mitochondrial ROS specifically could mitigate the AF-promoting effects of HPD.

Our study has limitations, and additional considerations are warranted. First, our HPD diet comprised 42% kcal protein, 44% kcal carbohydrate, and 13.1% kcal fat, which increased protein while decreasing carbohydrate content. Zhang et al. previously reported an increased incidence of AF in humans with lower carbohydrate intake over a median follow-up of 22 years [41]. However, it remains unclear if the risk of AF, varying with carbohydrate intake levels, shows significant differences in the short term, such as within a 5-year follow-up period [42]. Moreover, in the current study, the carbohydrate contributes to 44% of the total calories of HPD, which is close to the Institute of Medicine’s carbohydrate standard, constituting 45–65% of total caloric intake [43]. Therefore, the alterations observed in mice subjected to an HPD for 4 weeks, considerably shorter than the human study duration, are likely the direct result of the increased protein intake. Second, whether the increased percentage of protein in the diet or the duration of HPD feeding can aggravate AF should be addressed in follow-up studies. It should also be determined if HPD-mediated AF pathogenesis is reversible. Third, although the AIM2 inflammasome promotes abnormal SR Ca^2+^ leak, the precise molecular mechanisms require further extensive experimentation. Fourth, our study uses the whole atrial tissue for inflammasome activity. Therefore, we could not determine which cells are primarily responsible for the inflammasome activity, despite the lack of macrophage infiltration in the atria. Considering that AIM2 is known to be expressed in bone marrow-derived macrophages and other immune cells, and the bone marrow is the primary source of hemopoietic stem cells and immune cells [24,25], it is plausible that HPD may promote a low degree of inflammatory status within the bone marrow, thereby enhancing the inflammatory signaling in favor of atrial arrhythmogenesis. In future studies, single-cell transcriptome analysis would be helpful to dissect the intercellular interactions altered by HPD and inflammasome activation. A cardiomyocyte- or atrial-selective AIM2 knockout model would be beneficial to define the atrial myocyte-specific role of AIM2 in AF pathogenesis. Lastly, given that some inflammasomes such as AIM2 and NLRP3 share the same downstream pathway [44], studies using WT mice and mice deficient in caspase-1, ASC, or GSDMD are needed to uncover the optimal target to prevent AF associated with the inflammasome activations.

## 5. Conclusions

Our study reveals that high-protein diets can promote a substrate for AF development in mice. The AIM2 inflammasome serves as a mechanistic link between high-protein diets and AF pathophysiology. The activation of the AIM2 inflammasome in response to high-protein diets is mediated by dsDNA derived from stressed mitochondria. Targeting AIM2 might constitute a novel therapeutic approach against AF.

## Data Availability

The detailed experimental materials, methods, and data supporting the findings of this study are available within the article and its Supplemental Material. Raw data are available from the corresponding author upon reasonable request.

## Supplementary Materials

The following supporting information can be downloaded at: https://www.mdpi.com/article/10.3390/cells13020108/s1, Table S1. ECG parameters in wildtype (WT) and *Aim2^−/−^* mice. Table S2. Echocardiography parameters in WT and *Aim2^−/−^* mice 4-week feeding of NC or HPD. Figure S1. RT-PCR analysis of *Aim2* transcript in atrial tissue of WT and *Aim2^−/−^* mice. Figure S2. Incidence of pacing-induced AF in WT and *Nlrp3^−/−^* mice with NC or HPD feeding. Figure S3. High-protein diet improved glucose metabolism mice. Figure S4. Unchanged left ventricular diameters and anterior wall thicknesses. Figure S5. Unchanged protein level of macrophage marker. Figure S6. Quantification of mitoSOX intensity.
