# Supplementary material for: A High-Protein Diet Promotes Atrial Arrhythmogenesis via Absent-in-Melanoma 2 Inflammasome
Source: Cells. 2024 Jan 5;13(2):108. doi: 10.3390/cells13020108 (PMC10814244; doi:10.3390/cells13020108)
Supplement: Supplementary file 1 [file cells-13-00108-s001.zip › cells-2804173-supplementary.pdf]

Supplementary Material

A High-Protein Diet Promotes Atrial Arrhythmogenesis via Absent-in-Melanoma 2 Inflammasome

Jia Song, Jiao Wu, Dexter J. Robichaux, Tingting Li, Shuyue Wang, Maria J Arredondo Sancristobal, Bingning Dong, Dobromir Dobrev, Jason Karch, Sandhya S Thomas, Na Li

Supplementary Tables

Table S1. ECG parameters in wildtype (WT) and *Aim2*$^{-/-}$ mice

	WT-NC (n=14)	WT-HPD (n=18)	*Aim2*$^{-/-}$-NC (n=5)	*Aim2*$^{-/-}$-HPD (n=14)
HR (bpm)	483 ± 19	546 ± 12*	516 ± 22	489 ± 14$^{\#}$
PR (ms)	39.5 ± 0.89	36.8 ± 0.63*	38.5 ± 1.30	38.8 ± 0.99
QRS (ms)	9.4 ± 0.22	9.2 ± 0.25	9.7 ± 0.50	9.5 ± 0.33
QTc (ms)	17.0 ± 0.60	17.8 ± 0.30	17.2 ± 0.61	17.4 ± 0.55
cSNRT100 (ms)	1.3 ± 0.10	1.1 ± 0.06	1.3 ± 0.18	1.0 ± 0.07
cSNRT90 (ms)	1.3 ± 0.10	1.2 ± 0.08	1.4 ± 0.19	1.1 ± 0.10
AERP100 (ms)	41.7 ± 2.18	36.8 ± 1.02	39.2 ± 3.38	42.2 ± 2.20
AVW (ms)	76.0 ± 1.61	69.4 ± 1.00*	76.4 ± 1.83	72.2 ± 0.83
AVNERP100 (ms)	51.9 ± 1.64	45.2 ± 1.29*	50.0 ± 2.90	50.4 ± 1.61

HR, heart rate; SNRT, sinus node recovery time; AERP, atrial effective refractory period; AVW, atrioventricular Wenckebach; AVNERP, atrioventricular node effective refractory period. *P<0.05, WT-NC vs WT-HPD; $^{\#}$P<0.05, *Aim2*$^{-/-}$-HPD vs WT-HPD. P-values were determined by Welch ANOVA with Dunnett's T3 multiple comparisons test in HR and Kruskal-Wallis with Dunn's multiple comparisons test in PR, AVW, and AVNERP100.

Table S2. Echocardiography parameters in WT and *Aim2*$^{-/-}$ mice 4-week feeding of NC or HPD.

	WT-NC (n=7)	WT-HPD (n=12)	*Aim2*$^{-/-}$-NC (n=7)	*Aim2*$^{-/-}$-HPD (n=9)
EF (%)	60.2 ± 1.01	67.9 ± 2.90	58.7 ± 2.03	64.6 ± 3.48
CO (mL/min)	22.9 ± 2.25	20.1 ± 0.90	18.64 ± 0.86	20.2 ± 1.00
ESD (mm)	2.73 ± 0.13	2.26 ± 0.14	2.67 ± 0.13	2.48 ± 0.14
EDD (mm)	3.99 ± 0.16	3.60 ± 0.10	3.83 ± 0.12	3.82 ± 0.08
LVAWs (mm)	1.12 ± 0.10	1.29 ± 0.04	1.08 ± 0.07	1.27 ± 0.11
LVAWd (mm)	0.74 ± 0.04	0.81 ± 0.04	0.82 ± 0.04	0.88 ± 0.07
LVPWs (mm)	1.07 ± 0.07	1.40 ± 0.06*	1.15 ± 0.03	1.36 ± 0.09
LVPWd (mm)	0.71 ± 0.04	0.91 ± 0.05*	0.77 ± 0.02	0.92 ± 0.07

EF, ejection fraction; CO, cardiac output; ESD, left-ventricular end-systolic diameter; EDD, left-ventricular end-diastolic diameter; LVAWd, left-ventricular end-diastolic anterior wall thickness; LVAWs, left-ventricular end-systolic anterior wall thickness; LVPWd, left-ventricular end-diastolic posterior wall thickness; LVPWs, left-ventricular end-systolic posterior wall thickness. *$P<0.05$, WT-NC vs WT-HPD. P-values were determined by Ordinary one-way ANOVA with Welch ANOVA and Dunnett's T3 multiple comparisons test in LVPWs and LVPWd.

Supplementary Figures

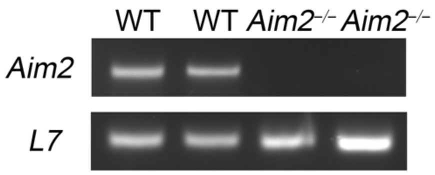

Figure S1. RT-PCR analysis of *Aim2* transcript in atrial tissue of WT and *Aim2*$^{-/-}$ mice.

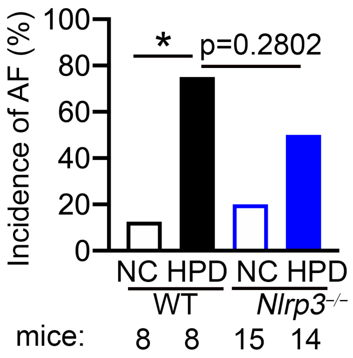

Figure S2. Incidence of pacing-induced AF in WT and *Nlrp3$^{-/-}$* mice with NC or HPD feeding.

*P<0.05. P-values were determined by Fisher's exact test.

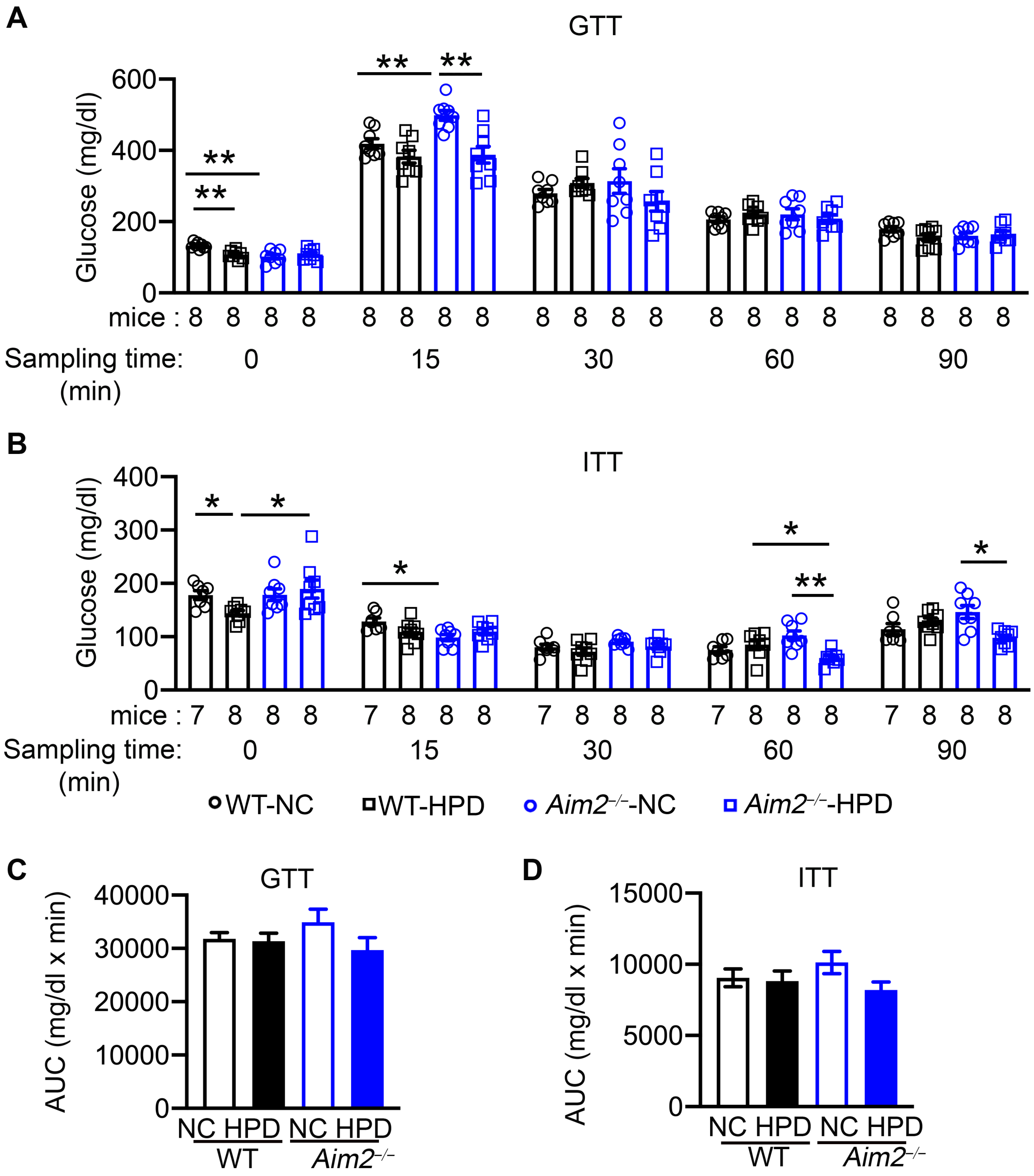

Figure S3. High-protein diet improved glucose metabolism mice. (**A**) Glucose levels during glucose-tolerance test (GTT). (**B**) Glucose levels during Insulin-tolerance test (ITT). (**C**) Quantification of the area under the curve (AUC) for glucose levels during GTT. (**D**) Quantification of AUC for glucose levels during ITT. *P<0.05, **P<0.01. P-values were determined by Welch ANOVA followed by Dunnett's T3 multiple comparison test in **A**, and Kruskal-Wallis with Dunn's multiple comparisons in **B**.

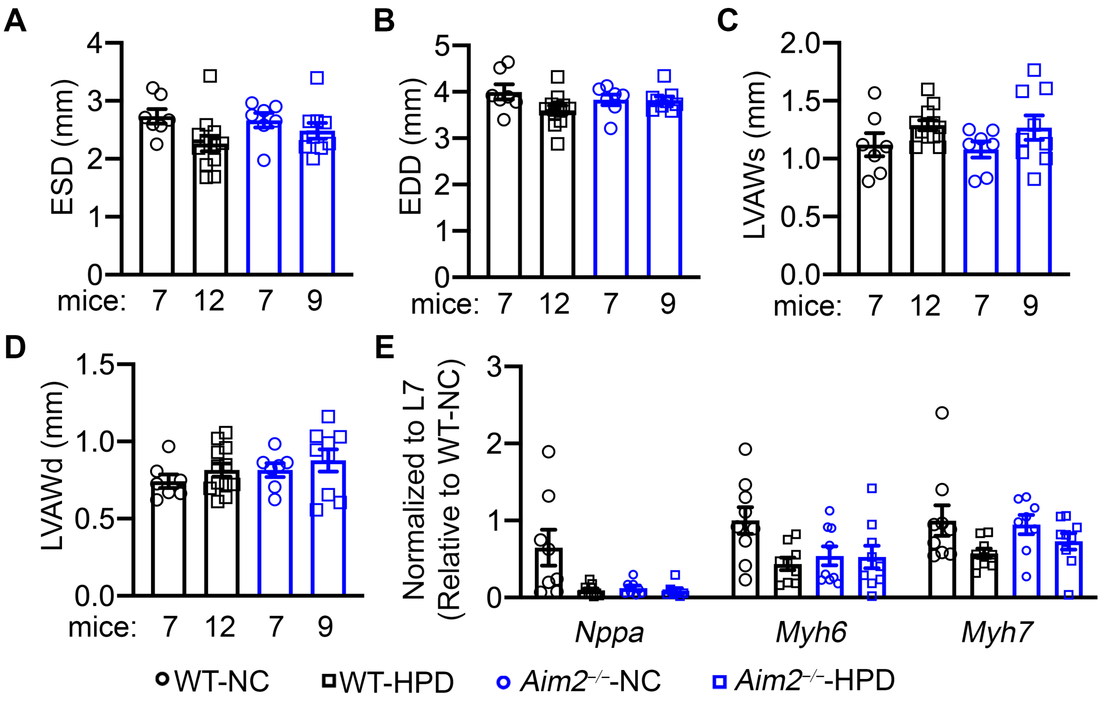

Figure S4. Unchanged left ventricular diameters and anterior wall thicknesses. (**A**) ESD. (**B**) EDD. (**C**) LVAWs. (**D**) LVAWd. (**E**) mRNA expression of hypertrophy markers (*Nppa*, *Myh6*, *Myh7*) in the ventricle tissue in WT and *Aim2*$^{-/-}$ mice with NC or HPD respectively.

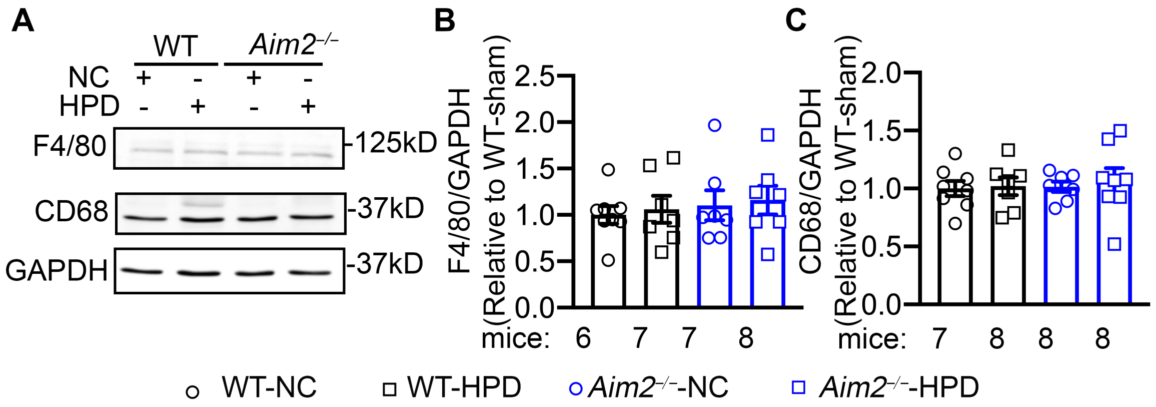

Figure S5. Unchanged protein level of macrophage marker. (**A**) Representative Western blots and quantification of **(B)** F4/80 protein level and **(C)** CD68 protein level in atrial tissues from WT and *Aim2*$^{-/-}$ mice with NC or HPD respectively.

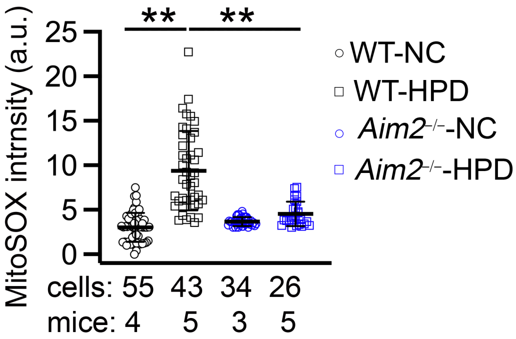

FigureS6. Quantification of mitoSOX intensity. **P<0.05. *P-values were determined by Nested one-way ANOVA followed by Sidak's multiple comparisons test.*
